# Case Report: Thoracoscopic secondary cytoreductive surgery for high-grade serous ovarian cancer

**DOI:** 10.3389/fsurg.2026.1737893

**Published:** 2026-03-05

**Authors:** Luigi Carlo Turco, Benedetta Alberghetti, Carlotta Francesca Cartia, Antonella Biscione, Giacomo Guidi, Filippo Maria Capomacchia, Andrea Droghetti

**Affiliations:** 1 Ovarian Cancer Center, Candiolo Cancer Institute, FPO-IRCCS, Candiolo, Italy; 2 Oncologica Thoracic Surgery Unit, Candiolo Cancer Institute, FPO-IRCCS, Candiolo, Italy; 3 Catholic University of the Sacred Heart, Rome, Italy

**Keywords:** minimally invasive techniques, ovarian cancer, recurrent ovarian cancer, SCS, thoracic surgery

## Abstract

The objective of this video is to show the feasibility of secondary cytoreductive surgery for recurrent high-grade serous ovarian cancer. The procedure targets recurrences in the cardiophrenic lymph nodes and the right diaphragm involving the right basal pleura. Using video-assisted thoracoscopy, the thoracic recurrences were dissected and removed. Minimally invasive thoracic secondary cytoreductive surgery proved to be safe and feasible and facilitated rapid post-operative recovery, ensuring timely access to subsequent second-line adjuvant chemotherapy.

## Introduction

Several studies demonstrated that secondary cytoreductive surgery (SCS) provides favorable oncological outcomes in patients with platinum-sensitive recurrent ovarian cancer, provided that complete tumor debulking is achieved (1, 2).

The objective of this video is to show the feasibility of a multidisciplinary approach to secondary cytoreductive surgery for a platinum-sensitive recurrence (PSR) in the right thorax. The procedure utilizes totally closed video-assisted thoracoscopy (TCVT) with CO_2_ insufflation, following an initial diagnostic laparoscopy.

## Patient information

The patient is a 60-year-old woman with an oligometastatic recurrence of high-grade serous ovarian cancer, following a platinum-free interval of 85 months.

## Timeline

**Figure 1:**
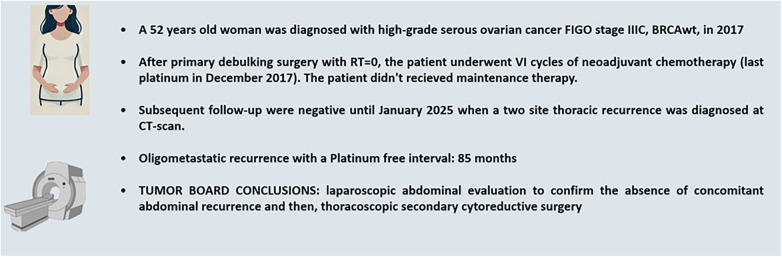
Patient clinical history timeline.

Figure 1 shows the timeline.

## Clinical findings and diagnostic assessment

CT imaging identified recurrence in the cardiophrenic lymph nodes and the right diaphragm, with involvement of the right basal pleura; PET-CT confirmed positive lymph nodes and right-diaphragm involvement.

An initial diagnostic abdominal laparoscopy was performed to rule out misdiagnosed miliary carcinomatosis not detected on the CT scan.

## Therapeutic intervention

Video-assisted thoracoscopy was then performed using two right-sided thoracic accesses: a primary access in the 8th intercostal space along the posterior axillary line and a secondary trocar in the sixth intercostal space along the mid-axillary line.

The thoracic recurrences were then dissected and removed. After removal of transdiaphragmatic disease infiltrating the muscle and the inferior margin of the lower lobe of the right lung, a diaphragm reconstruction was performed. For this reason, an atypical lung resection of the affected tissue was performed using a three-line stapler. The cardiophrenic lymph nodes were also removed. No intraoperative frozen pathology was used, as the radiological findings and macroscopic appearance of the tissue were highly suggestive of relapse.

## Follow-up and outcomes

Post-operative care included a 3-day hospital stay with basic clinical support. No complications were reported during or after the procedure. Blood loss was only 50 cc. Pathology confirmed the recurrence of high-grade serous ovarian cancer. After discharge from the hospital, the patient achieved full recovery within 4 weeks, and chemotherapy was initiated 36 days after surgery. No late complications were reported. Ca-125 levels were negative prior to starting adjuvant chemotherapy, and no post-operative imaging was performed.

Both early and late complications were graded according to the Clavien–Dindo classification.

## Discussion

Minimally invasive thoracic SCS is both safe and feasible and facilitated rapid post-operative recovery, ensuring timely access to subsequent second-line adjuvant chemotherapy. Since the time to chemotherapy is a critical factor in this setting, minimally invasive surgery (MIS) plays a key role in post-operative recovery by improving pain control and shortening hospital stay; when feasible, MIS should always be prioritized over open thoracotomy.

A diagnostic laparoscopy should always be considered to confirm the absence of concomitant disseminated abdominal disease.

In cases of oligometastatic, platinum-sensitive recurrence of ovarian cancer, SCS should be considered based on standardized patient selection criteria, such as the AGO score (1) and the iMODEL score (2).

In selected patients, thoracic SCS is safe and feasible, leading to improved oncological outcomes (1, 2).

A recent study by Dr. Ryan M Kahn at Memorial Sloan Kettering Cancer Center demonstrated that thoracoscopic cytoreductive surgery is safe and feasible even in the primary debulking surgery (PDS) setting. In addition, if this procedure is necessary to achieve complete gross resection, thoracoscopic cytoreductive surgery significantly impacts patient prognosis (3). In that study, only 31 patients (17%) experienced at least one grade ≥ 3 complication, with only 8 cases possibly related to intrathoracic cytoreduction (3).

In particular, when limited to cardiophrenic lymph node resection (CPLN), thoracoscopic dissection of pathological lymph nodes did not delay the initiation of adjuvant chemotherapy [the median time to chemotherapy was 40 days (range 19–205), which remains within the standard of care] (4).

A multidisciplinary evaluation and cooperation among oncologic surgeons, radiologists, and oncologists is crucial for proper patient selection and treatment in this setting. Surgical indications for such niche patient populations must be carefully evaluated, and this approach is not applicable to all thoracic recurrences in high grade serous ovarian cancer (HGSOC).

## Strengths and limitations

The strength of the procedure performed lies in its minimally invasive approach and the achievement of complete gross resection. In fact, minimally invasive surgery allowed the patient to quickly recover with minimal post-operative pain, ensuring that the patient remained on schedule for the initiation of adjuvant chemotherapy.

The main limitation of this surgical approach is the requirement for both abdominal and thoracic procedures, even when only a diagnostic laparoscopy was performed in the abdomen. In fact, this can be challenging, carrying an increased risk of morbidity in patients with a history of aggressive abdominal surgery for ovarian cancer. CT scan may not be sensible enough and can be false negative for miliary carcinomatosis. Diagnostic laparoscopy before thoracic surgery completely exclude abdominal disease and confirms the indication to thoracic secondary cytoreductive surgery. In addition, appropriate patient selection remains a challenge and is not entirely reliable, as preoperative imaging is often insufficient to detect miliary carcinomatosis.

## Patient perspective

Surgical resection of the disease itself was important for the treatment of the relapse; in fact, the procedure had a positive psychological impact on the patient.

The quick post-operative recovery and timely access to chemotherapy also gave more confidence to the patient, providing a more realistic perspective on achieving complete healing from the relapse.

## Data Availability

The original contributions presented in the study are included in the article/Supplementary Material, further inquiries can be directed to the corresponding author.
